# Supplementary material for: Massively parallel reporter assay–informed modeling improves prediction of context-specific enhancer–gene regulatory interactions
Source: Nucleic Acids Res. 2026 Jun 3;54(10):gkag554. doi: 10.1093/nar/gkag554 (PMC13232518; doi:10.1093/nar/gkag554)
Supplement: gkag554_Supplemental_Files [file gkag554_supplemental_files.zip › Supplementary legends.docx]

**Supplementary legends**

Supplementary 1. List of ENCODE accession numbers for all DNase-seq, H3K27ac ChIP-seq, and Hi-C data used in this study.

Supplementary 2. List of cell type–specific TF binding motif enrichment statistics for all TFs across K562, HepG2, and hiPSC cell lines.

Supplementary 3. Gene sets used for hypergeometric enrichment analyses of cell line–specific transcriptional programs, including Myc-responsive genes for K562, liver-specific genes for HepG2, and pluripotency-associated genes for the hiPSC line.

Supplementary 4. Table containing the E–G interaction networks for each cell line (K562, HepG2, and hiPSC). CRE coordinates, classes, target genes, and MPRabc scores are provided.
